# Was unterscheidet Führung in Präsenz vom virtuellen Raum?

**DOI:** 10.1007/s11613-022-00785-0

**Published:** 2022-09-05

**Authors:** Bernhard Schullan

**Affiliations:** Institut für Psychologie, Universität Kassel, Holländische Straße 36–38, 34127 Kassel, Deutschland

**Keywords:** Führung, Kommunikation, Virtueller Raum, Leadership, Communication, Virtual space

## Abstract

Die Diskussion über Führung in Präsenz und in der virtuellen Welt hat durch die Covid19 bedingte Verlagerung von Arbeitsplätzen in die Homeoffices neuen Schwung aufgenommen. Jedoch muss in die Diskussion miteinfließen, wie und wodurch Führung wirksam wird. Kommunikation auf sachlicher, affektiver und emotionaler Ebene ist das Bindeglied zwischen Führungskraft und Geführten. Aber gerade Kommunikation unterscheidet sich grundlegend in Präsenz und im virtuellen Raum. Das Kommunikationsmodell von Owen Hargie beschreibt detailliert die einzelnen Kommunikationsanteile in verbaler und nonverbaler Kommunikation. Somit können die einzelnen Kommunikationsanteile für die virtuelle, also technische Übertragung einzeln untersucht und bewertet werden.

## Abstract (en)

The discussion about leadership in presence and in virtual space has gained new momentum with the relocation of workplaces by Covid19 to home offices. However, the discussion must include how leadership becomes effective. Communication on a factual, affective and emotional level is the link between leader and employees. But communication in particular is fundamentally different in presence and in virtual space. The communication model of Owen Hargie describes in detail the individual communication parts in verbal and non-verbal communication. The individual communication parts for the virtual and technical transmission can be examined and evaluated individually.

Nach den anfänglich fast schon begeisterten Beschreibungen der ersten Monate der Pandemie, wie schnell und erfolgreich sich die Arbeitswelt auf die neuen Bedingungen des Arbeitens auf Distanz eingestellt hatte, tauchten in den späteren Monaten immer mehr Kritik, Enttäuschungen und Mangelbeschreibungen auf. Diese Veränderungen zu verstehen und nutzbar zu machen, muss ein großes Interesse für Berater:innen sein. Es ist davon auszugehen, dass sich dadurch in den kommenden Monaten und Jahren ein großer Markt an Beratungsbedarfen für Coaching, Supervision und Organisationsberatung ergeben wird. Im Besonderen kann man hier den Fokus auf die Entwicklung von Führungskräften legen, denn sie haben diese Veränderungen zu steuern, zu organisieren und letztlich zu verantworten. Was sind die Kernaspekte von Führung, die Wirksamkeit erzielen und damit für eine Unterscheidung von Führung in Präsenz oder im virtuellen Raum überhaupt relevant sind?

## Führungskompetenz als Kommunikationskompetenz

Im Kompetenzatlas für Führungskräfte von Erpenbeck und Heyse (2009) werden 64 Einzelkompetenzen unterschieden. Mehr als die Hälfte der im Kompetenzatlas verteilten Einzelkompetenzen benötigen zur Ausführung unmittelbar oder wenigstens mittelbar sehr hohe kommunikative Fähigkeiten (vgl. Gessler und Sebe-Opfermann 2016, S. 172). Dies unterstreicht einmal mehr die häufige Aussage: Kommunikation ist das Mittel der Führung.

„Psychodynamische Konzepte über die inneren Prozesse, die Dynamik von Organisationen, haben ihren Ursprung in der Theorie der Psychoanalyse“ (Lohmer und Giernalczyk 2012, S. 7). Bezieht sich diese Betrachtung zuerst auf einzelne Personen und ihre inneren Instanzen, so lässt sich das Konzept auch auf Gruppen und Organisationen anwenden (Lohmer 2008, S. 18). Alle handelnden Personen innerhalb einer Organisation tragen zur inneren Dynamik von Gruppen, Teams, Subsystemen und letztlich der ganzen Organisation bei (ebd., S. 8). Nach diesem Ansatz sollen die handelnden Personen in einer Organisation, sowohl Führungskräfte als auch die Geführten, in ihrer Ganzheit erfasst und betrachtet werden. Die Akteure sind letztlich die gleichen wie die Elemente der Systeme, Subsysteme, Umwelten in der Systemtheorie. Jedoch werden den Menschen grundsätzlich emotionale Tiefen mit Ängsten und psychosozialen Abwehrmechanismen zugestanden. Die in der Psychodynamik verwendeten Modelle gehen davon aus, dass den handelnden Personen ihre eigenen Ängste und die daraus folgenden Widerstände und Abwehrhandlungen weitgehend unbewusst sind.

Das psychodynamische Modell erweitert die Kommunikationsbedarfe in der Führung in emotionale und affektive Tiefen, die den Geführten, oft aber auch den Führungskräften selbst, nur schwer zugänglich sind oder gar im Unbewussten verborgen bleiben. „Jede Führungskraft hat neben ihren sachbezogenen Funktionen (Strategie, Management, strukturieren und entscheiden) in hohem Maße auch beziehungsorientierte und psychologische Funktionen (Leadership, Motivation, Bindung)“ (Giernalczyk et al. 2012, S. 41). Dabei ist es für die Führungskraft wichtig zu wissen, dass sie von den Geführten nicht nur als Person mit ihren Eigenschaften wahrgenommen wird, sondern immer auch mit ihren Rollen in der Organisation, die teilweise unbewusste psychologische Bedürfnisse auslösen oder provozieren.

Wenn es der Führungskraft bewusst ist, hilft es ihr, ausreichend Rollendistanz zu wahren. „Die Rollendistanz hilft, Zuschreibungen, Wahrnehmungen, Wertungen und Rückmeldungen nicht nur persönlich, sondern als Spiegelung von Rolle und Funktion zu nehmen und einzuordnen. […] Führungskräfte aktivieren aufgrund ihrer Rolle, in der sie Weisungen geben können, eine reale und fantasierte Macht über die Mitarbeiter, haben aber auch, weil sie über übergeordnete Informationen verfügen, Abhängigkeitseinstellungen bei ihren Mitarbeitern – ob sie wollen oder nicht. Ähnlich wie Kinder gegenüber Eltern tragen Mitarbeiter auch Schutzbedürfnisse, Wunsch und Bestrafungsfantasien an ihre Vorgesetzten heran“ (ebd., S. 41 f.).

In diesem aus dem Unbewussten gesteuerten Austausch von Emotionen liegen wertvolle Hinweise und Informationen für die Führungskräfte. Sie müssen jedoch willens und in der Lage sein, diese Informationen entsprechend aufzunehmen und zu verarbeiten (Burkhardt 2013, S. 406). Im psychodynamischen Verständnis wurde dafür von Wilfred Bion (1990) das Container-Contained-Modell und seine Bedeutung für Führungskräfte und Berater vorgestellt. Für diesen Artikel ist dabei interessant, dass das Container-Contained-Modell, bzw. das Containment, als Kommunikationsmodell bzw. als Kommunikationsprozess verstanden werden kann.

Zusammenfassend geht es also darum, in der interpersonellen Sensitivität das Befinden und Anliegen von Geführten, z. B. aus deren Mimik und Gestik und den situativen Bedingungen und Normen, zu erschließen. In der Führungssituation geht es dabei um eine ausgeglichene Balance der interpersonellen Sensitivität und der eigenen Selbstwahrnehmung. Dabei ist der Kontext sehr wichtig, in dem Kommunikation gelingen soll.

## Kommunikationsmodell von Owen Hargie

„In Teams fördert kommunikative Kompetenz beispielsweise effiziente Verständigung. Konflikte können auf diese Weise frühzeitig erkannt und konstruktiv gelöst werden. Besondere Herausforderungen stellen sich an die Kommunikation in Teams, wenn Kommunikation virtuell erfolgt und nonverbale Signale dadurch reduziert sind“ (Erler et al. 2012, S. 256). Dieser Hinweis auf Unterschiede bei virtueller Kommunikation soll nun genauer betrachtet werden. Das Kommunikationsmodell von Owen Hargie (1997) schlüsselt sehr detailliert einzelne Kommunikationsanteile oder -kanäle in auditive, visuelle und taktile Anteile auf. In Hargies Modell ist dabei auch die Kompetenz der kommunizierenden Personen im Fokus, die einzelnen Kanäle bedienen zu können. Gleichzeitig ist diese differenzierte Betrachtung der einzelnen Kanäle bestens geeignet, für jeden einzelnen Kanal oder Anteil eine technische Beschreibung und Bewertung durch den virtuellen Raum vorzunehmen. Damit kann das Modell von Hargie durch ein technisches Kommunikationsmodell ergänzt werden.

### Grundlagen

Die einzelnen Elemente des Kommunikationsmodells sind bei Hargie in sechs Komponenten für qualifizierte zwischenmenschliche Interaktion gegliedert (vgl. Schütz und Röhner 2020, S. 54): (1) Merkmale der Person und Merkmale der Situation (Person-Situation-Kontext), (2) Ziel(-e), (3) Vermittelnde Prozesse, (4) Antwortverhalten, (5) Feedback, (6) Wahrnehmung.

Zu den *Merkmalen der Person und den Merkmalen der Situation* zählen das (Vor‑) Wissen, ihre Motive, die Einstellungen mit kognitiven und affektiven Aspekten und einem aus dem Unbewussten gespeisten Verhalten. Weiter gehören die verschiedenen Persönlichkeitseigenschaften, die Emotionen, das Alter, das Geschlecht zu personalen Eigenschaften, die die Art und Weise von Kommunikation beeinflussen. Neben Personenfaktoren bestimmen auch Situationsfaktoren den Rahmen eines Kommunikationsprozesses. Schlüsselmerkmale einer Situation sind die Zielstruktur, die Rollen, die Umgebung, Sprache und Sprechweise (ebd., S. 71).

*Vermittelnde Prozesse* werden in kognitive und affektive Prozesse unterschieden. Die kognitiven Prozesse sind nötig, um einen Kommunikationsprozess als Empfänger überhaupt verstehen und einordnen zu können, um eine geeignete Antwort geben zu können. Dazu gehört die semantische Enkodierung, die Organisation der Informationen, die Speicherung und der Abruf von Informationen. Daraus entstehen schlussfolgernde Prozesse, um eine Antwort generieren zu können. Gleichzeitig steuern affektive Prozesse das Verhalten und beeinflussen damit die Entscheidungen. Sie färben sie sozusagen ein (ebd., S. 76).

Beim *Antwortverhalten* geht man davon aus, dass die antwortende Person einen Plan und ein inhaltliches Ziel entwickelt hat, die mit der Antwort umgesetzt werden sollen. Jedoch kann es während der Antwort dazu kommen, dass der Plan fehlerhaft umgesetzt wird und die Antwort dadurch verfälscht. Man spricht von Fehlhandlungen oder Auslassungsfehlern. Gelingende Kommunikation wird dadurch erheblich erschwert.

Im Laufe der Kommunikation erhält die kommunizierende Person internal und external übermitteltes Feedback. *Internales Feedback* entsteht durch eigene Körperwahrnehmungen, Herzklopfen, Schweißausbrüche, Müdigkeit und Erschöpfung oder ähnliche körperliche Reaktionen. In der virtuellen Welt kann ein besonderes internales Feedback dadurch entstehen, dass eine kommunizierende Person während der Videokonferenz ihr eigenes Videobild beobachtet und interpretiert. Dieses sehr spezielle Feedback wird jedoch oft als irritierend und ablenkend empfunden. Konstruktiver Umgang damit sollte daher geübt werden, um eine bessere Selbststeuerung der eigenen visuellen Botschaften zu erreichen.

*External vermitteltes Feedback* wird visuell, auditiv und taktil aus unserer Umgebung aufgenommen. Während der Interaktion verfolgen alle Teilnehmenden bestimmte Ziele und versorgen gleichzeitig durch das, was sie sagen und tun, ihr Gegenüber mit Informationen, die diesem wiederum als Feedback dienen. So kann man z. B. als *visuelles Feedback* aus Mimik und Gestik des Gegenübers Schlussfolgerungen über dessen Einstellung zum Thema schließen. Jedoch kann ein visuelles Feedback sehr leicht fehlinterpretiert werden, indem z. B. ein Stirnrunzeln des neuen Kollegen als Kritik an der eigenen Aussage verstanden wird, obwohl dieser lediglich sehr interessiert zuhört und mitdenkt (ebd., S. 78). Im Gegensatz zum realen Raum, in dem die Übertragung der visuellen Information in Lichtgeschwindigkeit erfolgt, kann die Übertragung im virtuellen Raum durchaus einzelne Sekunden benötigen. So entsteht möglicherweise eine große Unsicherheit, auf welchen Augenblick oder welche Situation das Feedback gesendet wurde. Im schlimmsten Fall geht das Feedback verloren oder wird falsch interpretiert.

*Auditives Feedback* kann sehr wirkungsvoll als kurze verbale Zustimmung oder Ablehnung gesendet bzw. empfangen werden, ohne die sprechende Person dadurch zu unterbrechen oder ihr direkt ins Wort zu fallen. Anderes paraverbales Feedback oder paralinguistische Phänomene sind ebenfalls geläufig (Watzlawick et al. 2017, S. 58). Der große Vorteil dieser Art von auditivem Feedback ist, dass es während des Sprechens der anderen Person gegeben werden kann, ohne sie zu unterbrechen. Ein positives Feedback in dieser Art führt zu einer Bestätigung des gesendeten Inhalts und der Vergewisserung, dass der sprechenden Person eine hohe Aufmerksamkeit geschenkt wird. Gerade in schwierigen Kommunikationssituationen zwischen Führungskraft und Geführten ist diese Form von Feedback sehr wirksam. Kommunikation in entsprechender emotionaler Tiefe kann dadurch gut gehalten und gestützt werden. In der Kommunikations-Situation entsteht eine intensive Bindung; hohe Konzentration, Aufmerksamkeit und Wertschätzung können so signalisiert werden. In einer Präsenz-Situation wird man einen geeigneten Raum und Rahmen wählen, um diese Intensivität, Konzentration und Bindungsmöglichkeit zu sichern.

Im virtuellen Raum ist die Möglichkeit, während des Zuhörens auditives Feedback zu geben, deutlich eingeschränkt. Die heute üblichen auditiven Kommunikationsverfahren stellen keine echt getrennten und gleichzeitigen Audiokanäle für Senden und Empfangen mehr zur Verfügung. Nur das ganz alte analoge Telefon hatte noch wirklich getrennte Audiowege, die gleichzeitig senden und empfangen konnten. Die heutigen Verfahren schalten den Sende- bzw. den Empfangsweg sehr schnell hin und her, sodass der Eindruck von Gleichzeitigkeit entsteht. In Wirklichkeit wird versucht, das jeweils lauteste Signal vorrangig zu schalten. In der Praxis bedeutet dies, dass ein leises auditives Feedback bei der im selben Augenblick sprechenden Person nicht ankommen kann und damit verloren geht. Ein entsprechend lautes Feedback führt zu einer Unterbrechung der sprechenden Person. Sprechen und dazugehörendes Feedback müssen also zeitlich seriell hintereinander erfolgen, damit keine Anteile verloren gehen. „Anderen Personen angemessen und wirkungsvoll Feedback zu geben ist eine wichtige Kompetenz im Rahmen von Kommunikation, die überdies trainierbar ist“ (Schütz und Röhner 2020, S. 79).

### Sinneswahrnehmung

Eine eigene Dimension des Kommunikationsmodells ist die Wahrnehmung. Eine Wahrnehmungsebene ist die Möglichkeit der Sinneswahrnehmung, die ähnlich wie beim Feedback zwischen visueller, auditiver und taktiler Wahrnehmung getrennt betrachtet werden kann. In einer Präsenzsituation mit mehreren Menschen ist es nicht garantiert, dass alle Personen gleichmäßig gut wahrgenommen werden können. Die visuelle Wahrnehmung ist selektiv abhängig von Parametern wie Raumgeometrie, Sitzordnung, Beleuchtungsstärke im Raum, aber ebenso abhängig davon, welche Aufmerksamkeit und damit visuelle Fokussierung auf eine einzelne Person gerichtet ist. Die auditive Wahrnehmung ist ebenfalls selektiv. Das binaurale Hören, d. h. das selektive Richtungshören mit zwei gesunden Ohren, hilft zum einen, viele verschiedene Schallereignisse aus ihren verschiedenen Richtungen zu unterscheiden, aber gleichzeitig auch die selektive Konzentration auf eine sprechende Person zu richten (Zwicker 1982, S. 6; Bodden 1993, S. 66; Blauert und Braasch 2008, S. 96). Taktile Wahrnehmung ist im Führungsalltag selten und gering ausgeprägt.

Im virtuellen Raum sind die Wahrnehmungsmöglichkeiten in der Kommunikation sehr eingeschränkt. Das Videobild einer teilnehmenden Person an der Konferenz kann nur übertragen, was diese Person mit ihrer Kamera und ihrer Selbstinszenierung zulässt. Nachlässigkeit bei der Ausrichtung der Kamera, der Ausleuchtung des Gesichts usw. schränkt die Wahrnehmung deutlich ein. Leider funktionieren Videokonferenzsysteme nicht immer so stabil, wie das erforderlich wäre. Die Wahrnehmung wird dadurch erheblich beeinträchtigt. In vielen Videokonferenzen wird daher zur Stabilisierung der Videoübertragung der aktuell sprechenden Personen als „Etikette“ empfohlen und gebeten, dass alle anderen Teilnehmenden ihre Kamera ausschalten. Für den Kommunikationsprozess ist das eine deutliche Einschränkung und einseitige Priorisierung der visuellen Wahrnehmung.

Auch die auditive Wahrnehmung im virtuellen Raum ist durch die technischen Möglichkeiten der Endgeräte und Systeme im Vergleich zu Präsenztreffen enorm eingeschränkt. Ein Beispiel, wie Wahrnehmung im virtuellen Raum sogar verbessert werden kann, ist die konsequente Nutzung von Kopfhörern und geeigneten Headset-Mikrofonen. Bei richtiger Anwendung entsteht dadurch eine Audioübertragung, die alle sprachlichen Details (verbale, paraverbale) optimal übertragen kann. Ein eventuell akustisch schwieriger, z. B. halliger Raum kann damit entschärft werden. Akustisch entsteht eine „Nähe“ zwischen Mund der sprechenden Person und Ohr der zuhörenden Person, die eher in sehr persönlichen, intimen Settings üblich ist. Das kann ein Vorteil für emotionale, vertrauensvolle Kommunikation sein; durch den detaillierten auditiven Kommunikationskanal lassen sich im Sinne der Psychodynamik Übertragungen in breiter Ebene leichter ermöglichen. Eine Schattenseite kann darin gesehen werden, dass eine solche intime Nähe eher als unangemessene Distanzlosigkeit empfunden wird, die der realen Situation zwischen den Beteiligten nicht entspricht.

### Soziale Wahrnehmung

Die soziale Wahrnehmung ist ebenfalls selektiv. Sie wird beeinflusst von den Wissensstrukturen, den Erwartungen an die Kommunikations-Teilnehmenden und von den Attributionsprozessen der wahrnehmenden Person. Sie ist sehr ungenau und mit Vorurteilen behaftet (Schütz und Röhner 2020, S. 79). Ein gelungener Beziehungs- und Vertrauensaufbau zwischen Führungskraft und Geführten gibt nötige Sicherheit. Mit Blick auf die Übertragung der Kommunikation in die virtuelle Welt ist die Differenzierung der einzelnen Kommunikationskanäle in auditive, visuelle und taktile Anteile entscheidend.

#### Verbale Kommunikation

Kommunikation wird üblicherweise in verbale und nonverbale Kommunikation unterschieden. In der nonverbalen Kommunikation werden alle Elemente zusammengefasst, die entweder über den Tastsinn oder den Sehsinn wahrgenommen werden. Auch die Proxemik, also die Signale, die durch das Einnehmen einer bestimmten Distanz zueinander ausgetauscht werden, ist in erster Linie visuell wahrnehmbar. Verbale Kommunikation ist immer mit paraverbaler Kommunikation verknüpft. Hargie (1997) nennt Linguistik die gemeinsame Klammer von verbal und paraverbal. In der Sprache von Watzlawick zählt er zum „Material“ jeglicher Kommunikation keineswegs nur Worte, „sondern auch alle paralinguistischen Phänomene (wie z. B. Tonfall, Schnelligkeit oder Langsamkeit der Sprache, Pausen, Lachen und Seufzen) und Körperhaltung, Ausdrucksbewegungen (Körpersprache) usw. In seinem Verständnis Verhalten jeder Art“ (vgl. Watzlawick et al. 2017, S. 58). Oder: „Neben der verbalen Information sind stets simultan paralinguistische Signale präsent, wie Prosodie, Lautstärke, Tempo, Timbre der Stimme, und zwar gleichzeitig und kontinuierlich und mit einer eigenständigen Informationskomponente (so signalisiert der Sprecher durch prosodische Merkmale seiner Äußerung, ob er noch weiterfahren möchte oder ob er das Rederecht abgeben will)“ (Imhof 2003, S. 30).

Für das Kommunikationsmodell ist es von Bedeutung, dass auch der auditive Teil der Kommunikation simultan zwischen zwei oder mehreren Kommunizierenden stattfinden kann. Auch wenn eine Person die hauptsächlich Sprechende ist, also verbale und paraverbale Botschaften sendet, so reagieren die Zuhörenden simultan ebenfalls mit paraverbalen Lauten als Feedback und Aufmerksamkeits-Signalisation. Gelegentlich werden die paraverbalen Signale als „Telefonlaute“ bezeichnet (Schulz von Thun et al. 2003, S. 73). In einem Telefongespräch sind paraverbale Anteile die einzige Art, dem Sprechenden ein simultanes Feedback zu geben. In Fortbildungsseminaren für Mitarbeitende mit viel Kundenkontakt sind Übungen für „hörbares Zuhören“ Standard.

Das rein gesprochene Wort bezeichnet Watzlawick als „digitale“ Kommunikation. „Die Anleihe ist der vereinbarte Sprach- und Sprech-Code, der von Sender und Empfänger gleichmäßig beherrscht werden muss, damit das gesprochene Wort auch gehört werden kann“ (Schütz und Röhner 2020, S. 90). Zum vereinbarten Code gehört allerdings, dass die verbale Kommunikation immer abwechselnd stattfindet. Die Praxis ist bekanntlich oft anders. Diese Feststellung ist deswegen wichtig, weil im virtuellen Raum für simultane Sprache keine technische Toleranz besteht. In Präsenz kann eine gewisse Simultanität von zwei gleichzeitig sprechenden Personen toleriert werden, ohne dass verbale Information verloren geht. Watzlawick et al. (2017, S. 70) bezeichnen die Kommunikationsanteile, die ohne den vereinbarten Sprach-Code auskommen, als „analoge“ Kommunikation; im Mittelpunkt steht dabei, *wie* gesprochen wird. Die Unterscheidung für die Anwendung von digitaler oder analoger Kommunikation definieren sie: „Überall, wo die Beziehung zum zentralen Thema der Kommunikation wird, erweist sich die digitale Kommunikation als fast bedeutungslos“ (ebd., S. 73). Es sei daran erinnert, dass Watzlawick die Begriffe „digital“ und „analog“ mit ganz anderer Bedeutung benutzt, als es heute üblich ist.

In dem Führungskraft-Geführten-System sind Beziehung und Bindung eine wesentliche Voraussetzung für gelingende Führung.

Nach Watzlawick et al. (ebd., S. 74) hat „jede Kommunikation einen Inhalts- und einen Beziehungsaspekt …, so wird deutlich, dass die digitale und die analoge Kommunikationsweisen nicht nur nebeneinander bestehen, sondern sich in jeder Mitteilung gegenseitig ergänzen. Wir dürfen ferner vermuten, dass der Inhaltsaspekt digital übermittelt wird, der Beziehungsaspekt dagegen vorwiegend analoger Natur ist“. „Warum sind nonverbale Botschaften so wichtig? Die nonverbale Kommunikation fungiert bei diskrepanten Botschaften quasi als Warnsignal für die rezipierende Person. Bestehen Diskrepanzen, werden nonverbale Signale oft als die „vertrauenswürdigeren“ gesehen“ (Zuckerman et al. 1982, zit. n. Schütz und Röhner 2020, S. 93). Für die Fokussierung auf Kommunikation im Führungsalltag in Präsenz oder im virtuellen Raum ist der Kern dieser Aussagen, dass vor allem bei widersprüchlichen Botschaften zwischen verbaler und nonverbaler Kommunikation den nonverbalen mehr Vertrauen geschenkt wird.

#### Zuhören

Verbale Kommunikation ist nichts wert, wenn dem Sprechenden nicht gut zugehört wird. Zuhören wird als sehr aktiver Prozess verstanden, der verschiedene Funktionen erfüllen soll: Beim Zuhören richtet sich der Fokus auf die Botschaften der sprechenden Person; ihr werden dabei Signale der Aufmerksamkeit gesendet, die wiederum wertschätzend und positiv aufgenommen werden können. Diese Rückkopplung verbessert die Qualität der Kommunikation. Auch kritische Nachfragen zu den Inhalten der gesprochenen Botschaften signalisieren Interesse, Aufmerksamkeit und eine ehrliche Auseinandersetzung mit dem Gesagten.

Für gelingende Kommunikation ist ein offenes, unverstelltes Zuhören wichtig. Vorwissen, auch im Sinne von Vorurteilen, sowie eine verzerrte Personenwahrnehmung können offenem Zuhören hinderlich sein. Wenn in Führungssituationen Konflikte gelöst werden müssen, ist offenes Zuhören eine wichtige Voraussetzung. Zuhören ist kein passiver Prozess, der automatisch oder nebenbei erfolgt; es benötigt Ressourcen (z. B. Aufmerksamkeit), was Zuhören anstrengend und fehleranfällig machen kann (Imhof 2009, S. 278). Beispielsweise müssen Informationen über die verschiedenen Kanäle richtig (z. B. verbal und nonverbal) aufgenommen, verstanden und abgespeichert werden.

„Der Zuhörer trägt zur Steuerung der Interaktion bei, indem er dem Sprecher sein Zuhören signalisiert (*listening cues*), Ermutigung zum Weitersprechen vermittelt oder Sprecherwechsel initiiert, aber auch, indem er durch Rückfragen und Einforderung von Erklärungsbedarf die Koordination der Sprecher- und Zuhörerabsichten im Auge behält, um zu vermeiden, dass die Gesprächspartner ins Leere oder aneinander vorbeireden“ (Imhof 2003, S. 39). Im umgekehrten Fall können Zuhörende ebenso bewusst oder unbewusst Anzeichen für Unaufmerksamkeit senden. Beispiele dafür sind (Schütz und Röhner 2020, S. 122):

- fehlender Augenkontakt,
- Gesichtsausdruck, der nicht zum Gesprächsinhalt „passt“,
- Signale für Müdigkeit oder alternative Interessen (z. B. Gähnen, Lesen, auf die Uhr schauen).

Diese drei Anzeichen von Unaufmerksamkeit beim Zuhören sind visuelle Signale, die durch verschiedene Parameter in der virtuellen Welt verzerrt oder verstärkt werden. Für verbale Kommunikation ist es ein drastisches Signal, wenn die zuhörende Person der sprechenden ins Wort fällt. In Gesprächen im realen Raum ist dieses Verhalten schon störend und abwertend. Im virtuellen Raum hat dieses Verhalten eine viel dominantere und abwertende Signalisierung, weil es in der virtuellen Welt nicht möglich ist, dass zwei auditive Signale simultan übertragen werden können. Die eigentlich sprechende Person wird akustisch unterdrückt und von der widersprechenden Person „abgewürgt“. Zusätzlich signalisieren verzögerte oder unterdrückte paraverbale Signale und irritierende Nebengeräusche große Unaufmerksamkeit.

#### Nonverbale Kommunikation

Die verbale Kommunikation dient gerade in Führungssituationen zum Austausch der Botschaften auf der Sachebene. Die nonverbale Kommunikation transportiert vor allem Affekte und Emotionen. „Bei nonverbaler Kommunikation können Botschaften simultan in beide Richtungen gesendet werden […] – beispielsweise signalisieren beide Kommunizierende durch Blickkontakt wechselseitig Interesse, während verbale Kommunikation in der Regel sequenziell verläuft“ (ebd., S. 89). Diese Aussage mag für ein Treffen in Präsenz richtig sein, weil die simultane visuelle Wahrnehmung im realen Raum wirklich in Echtzeit stattfindet. Im virtuellen Raum ist aber durch die technische Verzögerung der visuellen Übertragung keine echte Simultanität gewährleistet. Wird von einer Stelle ein visuelles Signal gesendet, so kann es aus technischen Gründen erst deutlich verzögert an der anderen Stelle wahrgenommen werden. Erfolgt daraufhin eine entsprechende visuelle Antwort, so ist der Rückweg ebenfalls verzögert. In den heute üblichen Daten-Netzwerken ist aber diese Verzögerung nicht eindeutig vorhersehbar. So bleibt eine erhebliche Unsicherheit bei der nonverbalen Kommunikation. Die dazugehörende verbale Kommunikation muss aus technischen Gründen nicht zwangsläufig synchron zu den visuellen Botschaften sein. Dies kann dazu führen, dass die „richtige“ visuelle Antwort zum „falschen“ akustischen Zeitpunkt kommt. Die Verzögerung von Antworten im virtuellen Raum kann durchaus einzelne Sekunden dauern.

#### Körpersprache

Zum Überbegriff der Körpersprache werden Gesten, Kopfbewegungen, Körperhaltung, Augen- und Blickkontakt sowie der Gesichtsausdruck gezählt. Das umfasst den größten Bereich der visuellen Kommunikation. Die vier basalen Emotionen Wut, Freude, Trauer und Angst können sehr differenziert über Körpersprache visuell kommuniziert werden. In dieser Kommunikationsform liegt eine große Glaubwürdigkeit. Daher ist es für differenzierte Gespräche und Auseinandersetzungen in der Führungskommunikation wichtig, zur verbalen und paraverbalen Kommunikation die Sicherheit gebende Körpersprache zu dekodieren. Im virtuellen Raum stehen Videokonferenzen für visuell unterstützende direkte und mindestens bidirektionale Kommunikation zur Verfügung. Die technischen Einschränkungen sind jedoch erheblich und werden oft unterschätzt. Eine unidirektionale visuelle Kommunikation ist z. B. ein voraufgezeichnetes Video. Hier ist es ein Vorteil, dass die Audioinformation garantiert zur visuellen Information synchron bleibt.

*Gesten* sind eine funktionale visuelle Kommunikationsmöglichkeit. Sie sind definiert als Bewegungen der Gliedmaßen, vor allem der Hände und Arme. Mit Gesten können durch ihre Darstellungsfunktion Objekte und Handlungen dargestellt werden, wie z. B. die Geste des Telefonierens oder die erhobene Hand als Stopp-Funktion. Die Ausdrucksfunktion von Gesten kann Informationen über den körperlichen und emotionalen Zustand einer Person übermitteln.

*Kopfbewegungen* können in der visuellen Kommunikation eine Steuerungsfunktion übernehmen. So kann mit einem Kopfnicken ein Wechsel der sprechenden Person angekündigt werden. Diese Steuerungsfunktion wird z. B. in einem reinen Telefongespräch ohne Bild über Prosodie, die gezielte Betonung einzelner Silben in Länge und Tonhöhe, gesteuert. Kopfnicken oder Kopfschütteln ist in unserem Kulturkreis eine visuelle Darstellung von Zustimmung oder Ablehnung, Ja oder Nein.

*Gesichtsausdruck: *„Gesichtsausdrücke spielen eine wesentliche Rolle bei sozialen Interaktionen. Der Gesichtsausdruck beeinflusst beispielsweise, ob wir einer Person vertrauen oder ob wir sie etwa für schuldig befinden. […] Kleinste Veränderungen des mimischen Ausdrucks modifizieren die zu übertragende Botschaft. Der Gesichtsausdruck beeinflusst, wie eine Person von anderen wahrgenommen wird“ (ebd., S. 106). Die emotionalen Ausdrucksmöglichkeiten des Gesichtsausdrucks sind besonders variabel und können sehr differenziert eingesetzt werden.

Gesten, Kopfbewegungen und Gesichtsausdruck miteinander kombiniert erfüllen als Gebärdensprache eine vollständige, differenzierte visuelle Kommunikations-Möglichkeit ohne auditive Unterstützung. Für alle drei Elemente gilt aber, dass sie nicht universell in allen Kulturen und Situationen gleich interpretiert werden.

Eine wesentliche Konstante für die Wirksamkeit dieser visuellen Kommunikationskanäle ist die zeitliche Präzision, das Timing. Wird die Gebärdensprache nur in einer Richtung als Übersetzung für hörgeschädigte Personen eingesetzt, ist das tolerierbar. Steht aber die visuelle Kommunikation mindestens bidirektional und in enger Verknüpfung mit auditiven Inhalten, so kann bei üblichen Übertragungsverzögerungen in der virtuellen Welt aus der unterstützenden Kommunikation eine behindernde, weil verfälschende Kommunikation werden.

*Die Körperhaltung* kann unter anderem Aufschluss über die Emotionen und die Einstellungen der Kommunizierenden geben. Eine weitere besondere Funktion innerhalb der Kommunikation, die besonders im professionellen Umfeld und gerade in Führungsbeziehungen unbewusst geklärt wird, ist das Aushandeln des jeweiligen Status der kommunizierenden Personen. Übertragen auf den virtuellen Raum ist das Aushandeln des Status in Videokonferenzen schwierig, denn es fehlen die objektiven räumlichen Bezüge, die in einem Präsenztreffen zur Verfügung stehen. Die Deutung der Körperhaltung vor einer Videokamera kann in gruppendynamischen Prozessen sehr auffällig sein.

*Augenkontakt und Blickkontakt: *„Augenkontakt bezeichnet ein einseitiges Anschauen. Blickkontakt kann dagegen als wechselseitiger Augenkontakt verstanden werden. Beide Formen können verbale Kommunikation begleiten, können aber auch getrennt davon stattfinden. Sie übermitteln Absichten, regulieren die zwischenmenschliche Interaktion und können Gefühle ausdrücken. Dabei ist zu beachten, dass Augenkontakt und Blickkontakt je nach Kontext und Mimik ganz unterschiedliche Bedeutungen haben können und beispielsweise entweder Zuneigung oder aber Konfrontation beziehungsweise Dominanz ausdrücken“ (ebd., S. 105). Blickkontakt kann bei sensiblen Menschen Ängste auslösen. „Augenkontakt und Blickkontakt können wichtige Informationen für die soziale Wahrnehmung übermitteln und helfen, mentale Zustände, Überzeugungen und Emotionen vom Gegenüber einzuschätzen. Dies wiederum ist relevant für die Interaktion mit anderen Menschen“ (ebd., S. 106). In Videokonferenzen ist es selten möglich, echten Blickkontakt zueinander aufzunehmen. Daher ist die oben genannte Differenzierung zwischen Augenkontakt und Blickkontakt hilfreich. Zwar kann man in Videokonferenzen Gesichter und Augen der Teilnehmenden auf dem Bildschirm betrachten, also selbst Augenkontakt aufnehmen. In den meisten Fällen ist aber die eigene Kamera nicht an der identischen Position wie das betrachtete Augenpaar und in der verzögerten virtuellen Welt nicht ausreichend synchron.

*Proxemik* beschreibt das räumliche Distanz-Nähe-Verhalten von Personen zueinander in einem gemeinsamen Raum. Dies wirkt sich in einer Führungsbeziehung spürbar aus, wenn Führungskraft und Geführte gegenseitig die räumliche oder territoriale Nähe bestimmen. Im virtuellen Raum ist eine kontinuierliche Steuerung der Distanz-Nähe-Wahrnehmung kaum zu realisieren.

*Physische Charakteristika* spielen im Führungsalltag eine deutliche Rolle. So ergeben sich z. B. immer wieder neue Dresscodes in Organisationen für Männer und Frauen. Im virtuellen Raum hat die plötzliche Einführung von Videokonferenzen im Alltag des mobilen Arbeitens die Kleiderordnungen deutlich durcheinandergewirbelt. Im Vergleich zu Organisationen, in denen Führung schon immer über Distanz nötig war, sind die Kleiderordnungen stabil, der Branche angemessen und können natürlich nur für den im Videoausschnitt (Portrait) sichtbaren Bereich wahrgenommen werden. „Physische Attraktivität ist dabei von enormer Bedeutung, denn attraktive Menschen werden unter anderem als freundlicher, kompetenter und intelligenter eingeschätzt als weniger attraktive Menschen“ (Langlois et al. 2000, zit. n. Schütz und Röhner 2020, S. 110).

## Technik und Physik im realen und im virtuellen Raum

### Sprechen in einem realen Raum

Die Hörqualität in realen Räumen wird durch Umgebungslärm zusätzlich beeinflusst. Störgeräusche, sei es durch einen Rasenmäher durch ein offenes Fenster, eine laute Klimaanlage im Raum oder durch unruhiges Verhalten und Nebengespräche anderer Teilnehmender, überlagern das diffuse Schallfeld und machen das Hören ebenfalls deutlich schwerer (Zollner und Zwicker 2013, S. 200).

### Binaurales Hören

Das menschliche Hören ist bei gesunden Menschen in der Lage, die Richtung der Sprache, die gehört werden soll, zu erkennen. Dazu wertet das Gehör die unterschiedliche Lautstärkenintensität am linken und rechten Ohr und zusätzlich den zeitlichen Versatz der ersten eintreffenden Schallwelle am linken und rechten Ohr aus. Dazu sind beide Ohren gleichzeitig notwendig, daher nennt man diese Fähigkeit binaurales Hören. Das Hören und letztlich das Zuhören werden in der verbalen Kommunikation dadurch erheblich erleichtert.

Eine gesunde Hörfähigkeit erlaubt es, in der Kommunikation gleichzeitig zu sprechen und zu hören. Das ermöglicht es, während des eigenen Sprechens gleichzeitig wichtige Rückmeldungen von den Teilnehmenden zu empfangen. Para-linguistische Feedbacks können ohne Anstrengung gehört werden. Auch verbale Botschaften können gleichzeitig empfangen werden. Die sprechende Person kann die Botschaften aufnehmen und selbst entscheiden, ob sie den eigenen Redefluss unterbrechen möchte oder in einer anderen Form, z. B. durch Körpersprache, darauf reagieren möchte. Dies ist in einem realen Raum möglich. In akustisch schwieriger Umgebung eines Raumes werden leise paraverbale Botschaften schneller verdeckt (ebd., S. 200) als laute verbale Nachrichten. Nonverbale Kommunikation ist von der Raumakustik unabhängig. Im virtuellen Raum wird Audio grundsätzlich mono übertragen, die hilfreiche Unterstützung des binauralen Hörens ist damit ausgeschlossen.

### Kommunikation in Echtzeit im virtuellen Raum

Findet Kommunikation im virtuellen Raum statt, also mediengestützte Kommunikation, ist es aus technischer Sicht sinnvoll, die Medien in ihre technischen Übertragungssysteme zu unterscheiden. Die erste Gruppe sind die Medien, die eine gleichzeitige, gemeinsame Kommunikation parallel ermöglichen. Das sind Audioübertragungssysteme wie Telefon, Mobilfunk oder Audiokonferenzsysteme und visuelle Übertragungsmedien wie Videokonferenzanlagen. Die zweite Gruppe der Medien sind dazu geeignet, zeitunabhängige Kommunikation herzustellen. Dazu gehören z. B. E‑Mails, Chats und Blogs. Für diesen Artikel ist die Fokussierung auf die Medien gerichtet, die eine gleichzeitige Kommunikation, also gemeinsame Telefongespräche oder virtuelle Treffen mit Videobild ermöglichen sollen.

Die Qualität von Audioübertragungstechnik ist nicht nur durch ihre Frequenzbandbreite geprägt, sondern auch durch ihre Geschwindigkeit. Diese ist abhängig von der Datenverbindung und der Umschaltsteuerung der Teilnehmenden.

### Videogestützte Kommunikationstechnik

Videogestützte Kommunikationstechnik, also Videokonferenzen benötigen ein Vielfaches an Datenvolumen für den übertragenen Videoanteil im Vergleich zum Audioanteil. Für die Übertragungsgeschwindigkeit gilt das gleiche Prinzip wie bei reinem Audio. Die Verzögerung der Kommunikationsanteile nimmt durch die Videonutzung nochmal erheblich zu. „In nicht seltenen Fällen kann das Delay hier aber mehrere 100 ms betragen. In der Praxis stellt sich heraus, dass kommunizierende Personen sehr empfindlich auf diese hohen Delays reagieren, sich sehr oft ins Wort fallen und die natürliche Kommunikation verloren geht. Bis zu einem Delay von 300 ms kann man noch ganz gut kommunizieren, bei höherem Delay wird ein Gespräch jedoch stark beeinflusst“ (Baum 2003, S. 8).

### Was zeigt die Kamera?

Für eine möglichst gelungene nonverbale Kommunikation ist es entscheidend, was die Kamera von einer Person aufnehmen kann. Für die Kommunikation ist es wichtig, das Gesicht mit seinen verschiedenen Ausdrücken und seiner Mimik gut erkennen zu können. Ebenso sollten die Körperhaltung und die Gestik gut erkennbar sein. Das bedeutet, dass eine gute Portraitdarstellung den besten Blick auf eine Person ermöglicht. Die Umgebung spielt dabei eine wichtige Rolle. Zum einen sollte die Person gut und gleichmäßig ausgeleuchtet sein, damit auch Details der Mimik erkennbar werden; starkes Hintergrundlicht durch ein Fenster z. B. erzeugt genau das Gegenteil. Ebenso erschwert oft die Gestaltung der Hintergrundbilder in Videokonferenzen eine wahrheitsgemäße, authentische Übertragung der Videoinformation für die Gesprächsteilnehmenden.

Bei vielen Videokonferenzen wird ein Blickkontakt nicht nur durch die zeitliche Verzögerung verschlechtert, sondern ebenso durch die organisatorische Gestaltung des Arbeitsplatzes und der Handhabung der kommunizierenden Personen. Für Blickkontakt müssen die Augen auf die Videokamera gerichtet sein, damit das Gegenüber den Blick empfangen kann. Das bedeutet aber, dass die Augen nicht auf das zugehörige Videobild des Gegenübers blicken können. So entsteht bei beiden Teilnehmenden eine große Irritation. In einem realen Raum kann man je nach Sitzposition mehrere Menschen gleichzeitig im Blick haben und deren nonverbale Kommunikation beobachten. Die Anzahl der Teilnehmenden einer Videokonferenz, die im Blick sein sollten, hängt sehr von der Bildschirmgröße ab. Bei zu kleinen Videobildern ist eine gute Deutung der nonverbalen Kommunikation nicht mehr gegeben.

## Der Anspruch an Führung in Präsenz und im virtuellen Raum

Um eine Unterscheidung zwischen Führung im realen Raum und Führung im virtuellen Raum zu finden, ist ein gemeinsames Verständnis von Führung und ihrer Wirksamkeit notwendig. Führung besteht aus komplexen sozialen Interaktionen. Daher ist ein Grundstein von Führungserfolg der Aufbau und die Pflege von Vertrauen und Beziehungen zwischen der Führungskraft und den Geführten. Das wirksame Mittel dazu ist gelungene Kommunikation auf den verschiedenen kognitiven, affektiven und emotionalen Ebenen. Containment ist dazu ein sehr wirksamer und geeigneter, aber auch anspruchsvoller Kommunikationskanal im Führungskontext. Also werden dafür sehr hohe Anforderungen an die Kommunikationsmöglichkeiten gestellt.

Die Begegnungsmöglichkeiten zwischen Führungskraft und Geführten bzw. den Geführten miteinander unterscheiden sich beim Arbeiten in einem realen Raum bzw. an einem gemeinsamen Standort einer Organisation erheblich zum virtuellen Raum. Die anfängliche Begeisterung nach dem Beginn des ersten Lockdowns 2020 über das erfolgreiche mobile Arbeiten ist längst abgeflaut. Viele Mitarbeitende fühlen sich von ihren Führungskräften ebenso wie von den Kolleginnen und Kollegen vernachlässigt und abgehängt.

Führung im virtuellen Raum erfordert deutlich höhere Ansprüche an Kommunikationsfähigkeiten und -möglichkeiten als in Präsenz. Visuelle Übertragungen unterstützen die nonverbale Kommunikation, dabei gibt es zeitunkritische Anteile, die einfach zu lernen und zu üben sind. Die zeitkritischen Anteile transportieren schon wichtige affektive Kommunikationsanteile, die gut beachtet werden müssen. Quasi-simultane nonverbale Kommunikation ist im virtuellen Raum so gut wie ausgeschlossen. Gleichzeitig steckt dort ein großes Potenzial für Fehlinterpretationen der Kommunikation. Beratung sollte hier sensibilisieren und Alternativen aufzeigen.

Das erweitert den Beratungsbedarf für Führungskräfte und ihre Mitarbeitende in Entwicklungskonzepten, Schulungsmaßnahmen und Trainings auch in der richtigen Anwendung von virtuellen Kommunikations-Tools. Dabei muss ein Bewusstsein geschaffen werden, welche technischen Einschränkungen die Kommunikation behindern und wie sie durch eine geeignete Organisation kompensiert werden können. Dazu im Folgenden einige Beispiele und pragmatische Hinweise.

### (1) *Zufällige und absichtsfreie Begegnungen*

Begegnen sich Kolleg:innen in realen Räumen der Organisation, sei es auf dem Flur, am Kopierer oder in der Teeküche, oder besuchen sie sich am Arbeitsplatz im Büro, kann man davon ausgehen, dass dies immer in dem Bewusstsein geschieht, in ihrem Arbeitsumfeld zu sein. Dazu gehört auch, dass es jederzeit möglich ist, ihre Führungskraft zu treffen, sei es zufällig, geplant oder spontan, aber immer beruflich veranlasst. Solche Gespräche sind wichtig für den Aufbau und die Pflege einer persönlichen Beziehung zwischen Führungskraft und den Geführten. Im virtuellen Raum existieren solche absichtslosen Treffen nicht. Diese Treffen sind aber so wichtig, dass sie in der Zusammenarbeit kompensiert werden müssen.

Wenn sich durch den Arbeitsbezug regelmäßige Einzeltreffen nicht ergeben, müssen anstelle der zufälligen Begegnung verabredete Kontakträume geschaffen werden. Dies sind vereinbarte Telefongespräche oder Videoschalten, die keinen arbeitsbezogenen Grund haben, sondern nur zum „Miteinander reden“ verabredet werden. Das wichtigste ist dabei, dass der Kontakt der persönlichen Beziehungspflege dient und nicht den Arbeitsinhalten. Die Menschen können eher als Person und weniger in ihrer Rolle wahrgenommen werden.

### (2) *Absichtsfreie Begegnungen in Gruppen oder Teams*

Der Begriff absichtsfrei bedeutet hier, dass kein direkter arbeitsbezogener Anlass zu diesem Treffen benötigt wird, sondern das Treffen selbst ist die Absicht. Auch Gruppen und Teams leben gerne mit Ritualen. So wird in vielen Arbeitsgruppen, Führungsteams oder Fachteams ein tägliches Ritual gepflegt, das nur dazu dient, mit den anderen Teammitgliedern im Kontakt zu sein. So hat z. B. eine Führungskraft seit vielen Jahren jeden Morgen zur gleichen Zeit ihr Führungsteam in ihr Büro an einen Stehtisch eingeladen, um für 15 min eine Tasse Kaffee zu trinken und miteinander zu sprechen. Themen können der Fernseh-Krimi des letzten Abends sein, Erzählungen über die Kinder, eine neue Restaurant-Entdeckung oder andere private Themen. Die Begegnung ist frei von Hierarchie, und die einzelnen Rollen können in den Hintergrund rücken. Trotzdem ist bei Bedarf die Möglichkeit gegeben, noch ein besonderes Ereignis des letzten Arbeitstages zu erzählen, oder auf einen wichtigen kommenden Termin des Tages hinzuweisen.

Dieses Ritual hat mit der Covid-19-Pandemie eine wesentliche Veränderung erfahren. Die Mitglieder des Führungsteams haben sich unter Corona-Bedingungen an den ersten Tagen des Lockdowns in einer Videokonferenz zur gleichen Uhrzeit getroffen. Nach ein paar Tagen hatten sich jedoch die Assistenzkräfte bei der Führungskraft beschwert, dass sie nun keine Informationen mehr über die Themen bekämen, die in dem „Stehkaffee“ besprochen werden. Vor der Pandemie hatte sich das Ritual in seinem Wert und seiner Funktion so gut etabliert, dass jede Führungskraft danach ihrer Assistenz über das Treffen berichtet hatte. Die Lösung war daher sehr einfach. Seither nehmen alle Führungsteam-Mitglieder und ihre Assistenzkräfte jeden Tag für die eine Viertelstunde an dem virtuellen Treffen teil. Die Wertschätzung für dieses einfache Treffen ist so hoch, dass man sich persönlich und rechtzeitig im Chat entschuldigt, wenn man nicht teilnehmen kann. Gleichzeitig ist es eine geeignete Übungsmöglichkeit für Kommunikationsdisziplin: zuhören, ausreden lassen, gegenseitig Raum geben.

### (3) *Arbeitsbezogene, regelmäßige Einzeltreffen „Jour fixe“*

Im Führungsalltag ist es in Präsenzführung üblich, pro Woche ein festgelegtes persönliches Treffen zwischen Geführten und Führungskraft zu etablieren. Der Arbeitsbezug erfordert eine Tagesordnung, die von beiden Seiten vorbereitet werden muss. Denn dieses Treffen ist zeitlich begrenzt, in der Regel etwa eine Stunde. Zu Beginn des Treffens muss die Tagesordnung allen bekannt sein, damit gemeinsam die Zeiteinheiten für jedes Thema festgelegt werden können. In einem gut vorbereiteten Rahmen ist es auch im Führungsalltag möglich, themenbezogen zwischen handlungsorientierter Haltung und reflektierender Haltung für Containment zu wechseln.

Im virtuellen Raum ist ein solches Treffen schwieriger zu gestalten. Die Mechanismen sind im Prinzip ähnlich, jedoch sind viele Menschen im virtuellen Raum dazu verleitet, die kognitive Sachebene gar nicht mehr zu verlassen, um diszipliniert und erfolgsorientiert die Tagesordnung abzuarbeiten. Aber dabei bleiben die Emotionen, Affekte und Gefühle verborgen und können sich somit aufstauen. Daher ist es wichtig, dass die Führungskraft explizit z. B. einen Zeitraum für emotionale Themen reserviert und dies kommuniziert. Insgesamt kann das bedeuten, dass der Zeitbedarf pro mitarbeitende Person ansteigt. Durch regelmäßige Übung sollte sich das aber ausgleichen lassen. Auf lange Sicht gesehen ist es auf jeden Fall vorteilhaft, denn wenn sich die Emotionen im Verborgenen aufstauen und Konflikte eskalieren, ist der dann benötigte Zeitbedarf, um den Vertrauensverlust und andere Schäden zu beseitigen, erheblich größer.

### (4) *Arbeitsbezogene, regelmäßige Gruppen- oder Teamtreffen*

Bei Team- oder Gruppentreffen ist es traditionell üblich, dass die Führungskraft zusätzlich die Rolle der Moderation übernimmt. Grundsätzlich kann es hilfreich sein, wenn diese Rolle nacheinander von allen Teilnehmenden einmal übernommen wird. Teamtreffen verlangen in der Regel eine vorbereitete Tagesordnung. Eine Führungskraft sollte einordnen können, welcher Behandlungsmodus für welches Thema gewählt werden sollte. Das kann die kognitive Ebene der Systemtheorie sein, auf der unterschieden wird, ob es sich um ein einfaches, ein kompliziertes, ein komplexes oder gar chaotisches Thema handelt. Ein Thema kann auch einen gruppendynamischen Ursprung haben. Die Behandlung des Themas wird sich dadurch verändern. Auf der affektiven und emotionalen Ebene muss vielleicht eine psychodynamische Tiefe erreicht werden. Dazu muss das Team oder die Gruppe in einen anderen Modus wechseln.

Im virtuellen Raum gelten im Prinzip dieselben Regeln. Die Toleranz für Abweichungen davon ist jedoch deutlich niedriger. Dynamische Interaktionen, also laute und schnelle Gesprächsteile können im virtuellen Raum nicht abgebildet werden. Der Anspruch an entsprechende Disziplin der Teilnehmenden ist besonders hoch. Das kann dazu führen, dass sich einzelne Teilnehmende nur ungern beteiligen möchten und sich gedanklich zurückziehen. Gerade gruppendynamische Effekte können die Dynamik deutlich anheizen. Die Anforderungen an die Moderationsrolle steigen erheblich. Hat die Führungskraft die Moderationsrolle, kann sie sich durch ihre zugeschriebene Macht gegen dominante und auffallende Teilnehmende besser durchsetzen als andere gleichgestellte Teilnehmende bei dem oben beschriebenen rollierenden Moderationsverfahren. Ist während eines Termins ein Wechsel von handlungsfokussiertem zu reflexionsorientiertem Modus nötig, muss dieser Wechsel für alle Teilnehmenden deutlich nachvollziehbar markiert werden.

Im virtuellen Raum kann es sinnvoll sein, bei einer großen Teilnehmerzahl Diskussionen und Reflexionen auf aktive und passive Teilnehmende zu begrenzen. Dazu eignen sich Formate wie Fishbowl-Diskussionen oder ähnliche, bei denen die aktive Teilnehmerzahl bewusst eingeschränkt wird. Die aktiven Teilnehmer können am Bildschirm sichtbar dargestellt werden, die passiven, also nur beobachtenden Teilnehmenden schalten ihre Kamera aus. Es sollte nur sichergestellt werden, dass sich im Laufe der Zeit auch alle an dem Format beteiligen können. Ein Vorteil von manchen Videokonferenzsystemen gegenüber realen Treffen ist die schnelle Aufteilung der Teilnehmenden in verschiedene virtuelle Gruppen.

### (5) *Kommunikation mit einer großen Gruppe von Mitarbeitenden*

Die bisherige Betrachtung der Begegnungsmöglichkeiten bezog sich auf eine Führungskraft und die direkt von ihr geführten Personen. In jeder Organisation ist es immer wieder wichtig, dass sich auch die Menschen angesprochen fühlen, die nicht direkt von der oberen Führungskraft geführt werden. In Zeiten von Veränderungen und Umbruch innerhalb einer Organisation oder einer Bedrohung ist gute und ehrliche Kommunikation in die ganze Organisation essenziell. Aus psychodynamischer Perspektive muss eventuell die primäre Aufgabe der Organisation verändert werden und eine grundlegende Neuausrichtung der Organisationsstrategie erfolgen. Diese Bedingungen lösen in einer Organisation Ängste aus, die zu Abwehr oder organisationalem Fehlverhalten führen können. Daher ist auch sehr regelmäßige Kommunikation wichtig. Solche großen Treffen sind jedoch sehr aufwändig zu organisieren. Vielleicht muss erst ein geeigneter Raum organisiert werden, die Mitarbeitenden müssen ihre Arbeit unterbrechen und zu der Veranstaltung gelangen können. Wenn sich die Mitarbeitenden beteiligen sollen, steigt der Aufwand zusätzlich. Die Folge ist, dass solche wichtigen Treffen und Veranstaltungen zu selten durchgeführt werden.

Im virtuellen Raum liegt hier ein sehr großer Vorteil. Videokonferenzen mit vielen Teilnehmenden können schnell und effektiv organisiert werden. Alle können vom Büro-Arbeitsplatz oder von zuhause aus teilnehmen. Die effektive Teilnehmerzahl ist in der Regel größer als bei realen Treffen. Präsentationen können allen Teilnehmenden einfach zur autonomen Verwendung geschickt werden, Fragen und Anmerkungen der Mitarbeitenden können in einem Chat gesammelt und noch während der Veranstaltung beantwortet werden. Stimmungsabfragen per Chat oder anderen Tools können der Führungskraft ein viel ehrlicheres Bild über den emotionalen Zustand der Organisation vermitteln als bei realen Treffen, in denen oft die immer gleichen, schon bekannten Personen das öffentliche Wort ergreifen. Der wirksame Nutzen einer solchen virtuellen Veranstaltung ist gemessen am effektiven Zeitaufwand sehr hoch. Damit empfiehlt es sich, diese Veranstaltungsform gerade in schwierigen Zeiten regelmäßig zu nutzen.

## References

[CR1] Baum, H. (2003). VoIP und Videokonferenzen. In: Computer Supported Cooperative Learning (CSCL) Technologien und Anwendungen. https://pi4.informatik.uni-mannheim.de/pi4.data/content/courses/2003-ws/cscl_seminar/VoIP.pdf. Zugegriffen: 26. Okt. 2021.

[CR2] Bion WR (1990). Lernen durch Erfahrung.

[CR3] Blauert J, Braasch J, Weinzierl S (2008). Räumliches Hören. Handbuch der Audiotechnik.

[CR4] Bodden M (1993). Binaurales Hören und die Hörgeräte-Technologie der Zukunft. Audiologische Akustik.

[CR5] Burkhardt C (2013). Bewusster Umgang mit Übertragungsphänomenen als Management Soft Skill. Organisationsberatung, Supervision, Coaching.

[CR6] Erler S, Jaek A, Schütz A, Beutner E, Maas G, Neukirchner H (2012). Der Faktor Mensch in der virtuellen Produktentwicklung. Virtuelle Produktentwicklung.

[CR7] Erpenbeck J, Heyse V (2009). Kompetenztraining: Informations- und Trainingsprogramme.

[CR8] Gessler M, Sebe-Opfermann A, Müller-Vorbgüggen M, Radel J (2016). Kompetenzmodelle. Handbuch Personalentwicklung.

[CR9] Giernalczyk T, Lohmer M, Heimer C, Albrecht C, Giernalczyk T, Lohmer M (2012). Führung aus psychodynamischer Perspektive. Das Unbewusste im Unternehmen.

[CR10] Hargie O, Hargie O (1997). Interpersonal communication: A theoretical framework. The handbook of communication skills.

[CR11] Imhof M (2003). Zuhören: psychologische Aspekte auditiver Informationsverarbeitung.

[CR12] Imhof M (2009). Hören ist noch nicht zuhören. UGB-Forum – Fachzeitschrift für Gesundheitsförderung.

[CR14] Lohmer M (2008). Psychodynamische Organisationsberatung: Konflikte und Potentiale in Veränderungsprozessen.

[CR13] Lohmer M, Giernalczyk T, Giernalczyk T, Lohmer M (2012). Psychodynamik und Unbewusstes in Unternehmen. Das Unbewusste im Unternehmen.

[CR15] Schulz von Thun F, Stratmann R, Ruppel J (2003). Miteinander reden: Kommunikationspsychologie für Führungskräfte.

[CR16] Schütz A, Röhner J (2020). Psychologie der Kommunikation.

[CR17] Watzlawick P, Beavin JH, Jackson DD (2017). Menschliche Kommunikation: Formen, Störungen, Paradoxien.

[CR18] Zollner M, Zwicker E (2013). Elektroakustik.

[CR19] Zwicker E (1982). Psychoakustik.

